# One Year of Pandemic Learning Response: Benefits of Massive Online Delivery of the World Health Organization’s Technical Guidance

**DOI:** 10.2196/28945

**Published:** 2021-04-21

**Authors:** Heini Utunen, Maria D Van Kerkhove, Anna Tokar, Gillian O'Connell, Gaya M Gamhewage, Ibrahima Socé Fall

**Affiliations:** 1 Health Emergencies Programme World Health Organization Geneva Switzerland

**Keywords:** COVID-19, e-learning, massive open web-based courses, OpenWHO, pandemic, public health, web-based learning, World Health Organization

## Abstract

The World Health Organization (WHO) launched the first web-based learning course on COVID-19 on January 26, 2020, four days before the director general of the WHO declared a public health emergency of international concern. The WHO is expanding access to web-based learning for COVID-19 through its open-learning platform for health emergencies, OpenWHO. Throughout the pandemic, OpenWHO has continued to publish learning offerings based on the WHO’s emerging evidence-based knowledge for managing the COVID-19 pandemic. This study presents the various findings derived from the analysis of the performance of the OpenWHO platform during the pandemic, along with the core benefits of massive web-based learning formats.

## Introduction

The World Health Organization (WHO) launched the first web-based learning course on COVID-19 on January 26, 2020, four days before the director general of the WHO declared a public health emergency of international concern. The WHO is expanding access to web-based learning for COVID-19 through its open-learning platform for health emergencies, OpenWHO. Throughout the pandemic, OpenWHO has continued to publish learning offerings based on the WHO’s emerging evidence-based knowledge for managing the pandemic.

Several findings derived from the analysis of the performance of the OpenWHO platform during the pandemic are presented herein, with regards to the global reach of the courses [1], growth in the uptake of OpenWHO’s web-based learning resources [2], and trends in platform usage and the incidence of COVID-19 [3].

The course “Introduction to COVID-19” is hosted on the WHO Health Emergencies learning platform OpenWHO.org and the Pan American Health Organization’s Virtual Campus platform and has registered more than 1.15 million enrollments with versions in 40 languages, and versions in several more languages are in current production. As new evidence emerges, the course content is continuously updated to include the latest scientific knowledge and align with the WHO’s latest technical guidelines. The course has been revised 11 times since its’ launch. The “Introduction to COVID-19” course is currently available in Arabic, Chinese, English, French, Russian, Spanish, Amharic, Bengali, Dari, Esperanto, Fula, German, Hausa, Hindi, Hungarian, Igbo, Indian sign language, Indonesian, Kurdish, Latvian, Macedonian, Marathi, Oriya, Oromo, Pashto, Persian, Portuguese, Punjabi, Serbian, Somali, Swahili, Tetum, Telugu, Thai, Turkish, Vietnamese, Urdu, Yoruba, and Zulu.

**Figure 1:**
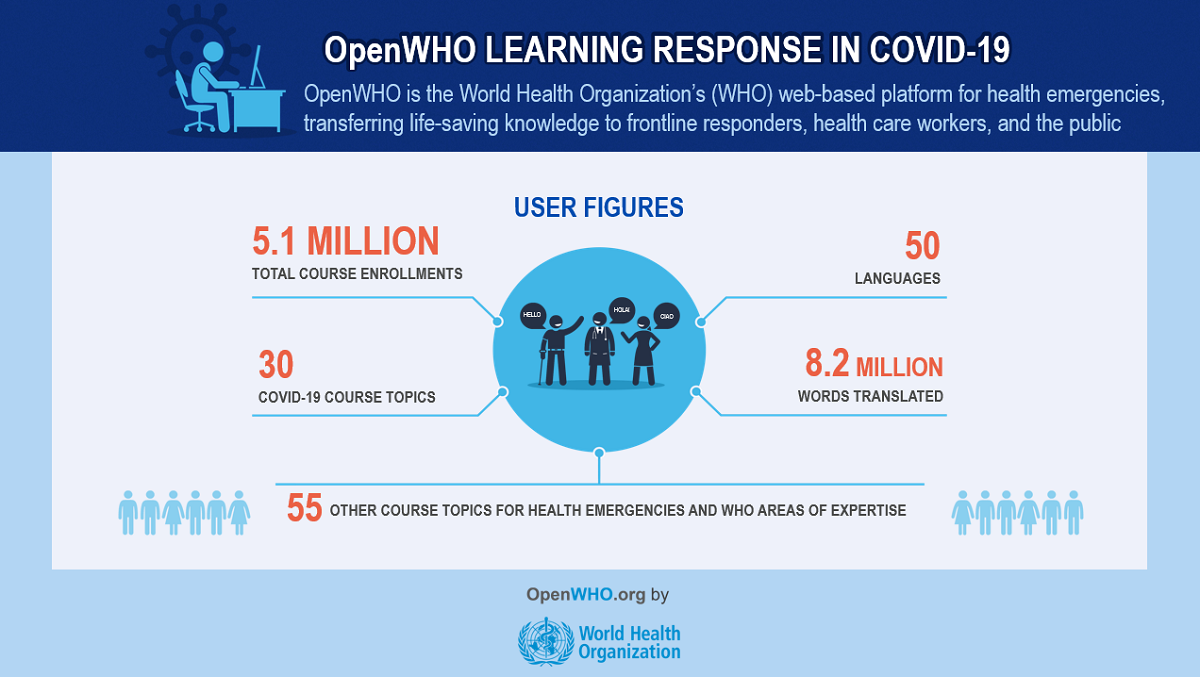
OpenWHO key figures as of March 2021.

As shown in Figure 1, by March 2021, the OpenWHO platform has encompassed 50 languages and 5 million course enrollments, of which more than 80% are related to COVID-19. OpenWHO has issued a total of 2.8 million certificates, half for completion and half for achievement, thus achieving an average course completion rate of 50% on the platform.

**Figure 2:**
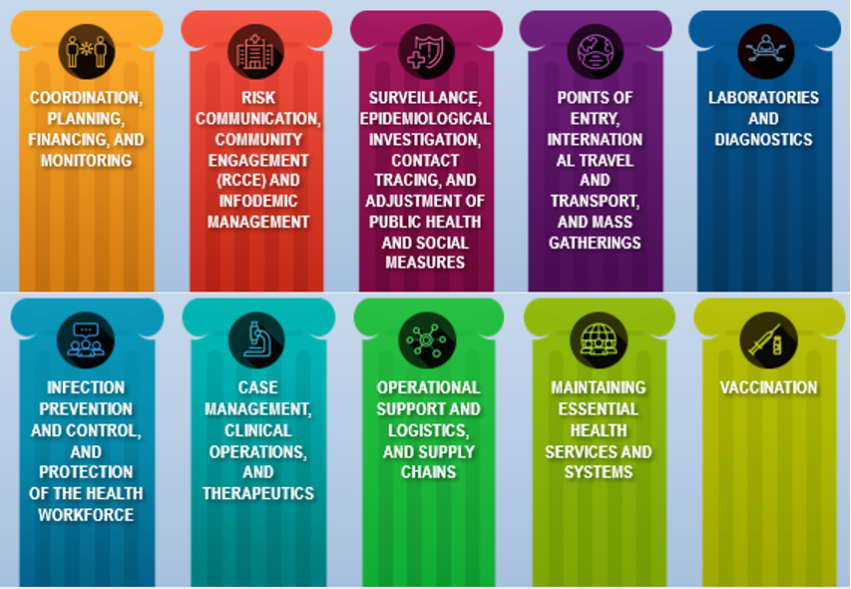
OpenWHO courses span all the intervention areas of the COVID-19 special preparedness and response plan.

Free training is available on 30 different COVID-19–related topics to support the COVID-19 response. These COVID-19–related courses cover the following topics: an introduction to COVID-19, clinical care, infection prevention and control, COVID-19 vaccination training, national deployment and vaccination planning, vaccine-specific knowledge resources, guidance on mask use, long-term care, clinical management, rehabilitation of patients with COVID-19, leadership in infection prevention and control, staying healthy and safe at work, country capacitation, treatment facility design, the Go.Data tool, personal protective equipment, hand hygiene, waste management, risk assessment for mass gatherings, occupational health and safety, eProtect predeployment training, country intra-action reviews, neglected tropical diseases in the pandemic context, COVID-19 risk communication, public health emergency operations centers, and other related topics (Figure 2).

## Core Benefits of the Massive Web-Based Learning Formats

**Figure 3:**
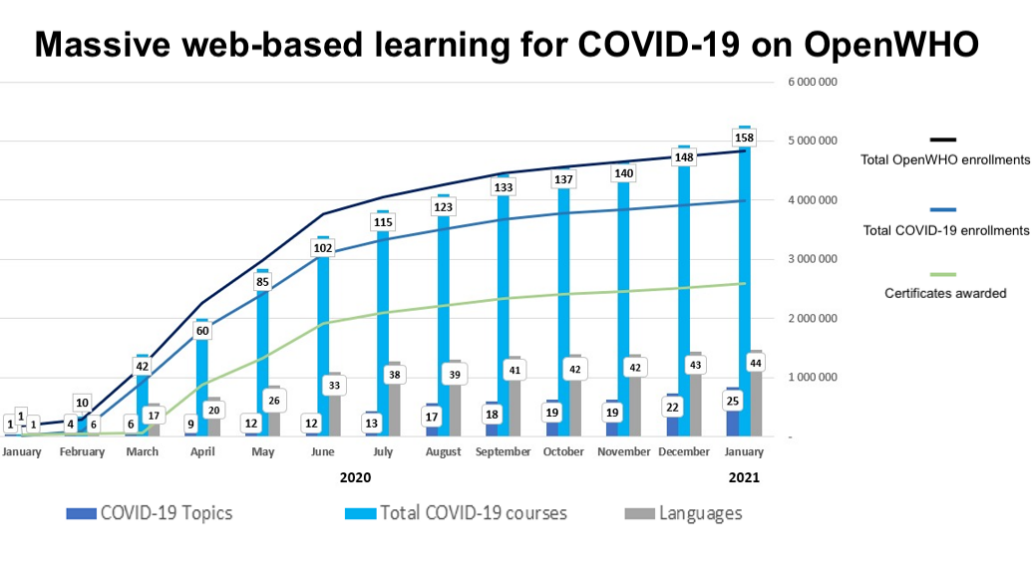
OpenWHO COVID-19 topics, languages, enrollments, and certificates from January 26, 2020 to January 26, 2021.

The COVID-19 pandemic has led to a significant and rapid increase in all forms of digital and web-based learning. The application of an equity lens to the web-based learnings offered by the OpenWHO platform (Figure 3) has yielded unprecedented access to the WHO’s knowledge and know-how during the current crisis. The following factors have led to the success of this unprecedented training and learning response in response to the current pandemic:

1. Equity: the design of learning activities is based on the principles of equity to health, supported by equity in access to education, and learning for health. Cost and digital barriers often inhibit those who most need knowledge from accessing it. The elimination of these barriers has been the fundamental premise of the WHO’s health emergency training response. Equitable access to critical health emergency knowledge helps provide core learning in the native languages of the most vulnerable populations and includes sign language.
2. Accessibility: web-based learning enables participants with even basic technology to access learning from almost anywhere in the world. OpenWHO courses are globally successful because they are free, self-paced, low-bandwidth adjusted, downloadable and portable, and available on any device. Offline options increase access even further.
3. Flexibility: self-paced mass web-based learning delivery enables individuals to learn at their own speed, at their preferred time, and in their preferred place. It builds on and provides for the learners’ preferences and availability.
4. Learner-centricity: user-friendly options allow individuals to choose formats specific to their learning needs and provide the basis for more customized “just-in-time” learning experiences and continuous, lifelong learning.
5. Quality: courses that are based on the latest scientific evidence and on WHO technical guidance and the use of adult learning techniques assure the quality of content and enhance learning.

## Literature Review and Discussion

Even though embedding impactful digital and web-based learning remains a challenge for all areas of knowledge [4-6], its effectiveness has already been demonstrated in medical education and beyond in times of distress [4-9].

The current COVID-19 crisis could be seen as a so-called “black swan moment” with regard to the training of health professionals and the need to examine the role of e-learning in particular [10-12]. The experience of OpenWHO in responding at scale and pace to the urgent need for high-quality and accessible web-based learning for COVID-19 has clearly and consistently demonstrated that the positive effects of digital learning are diverse and multiple and are relevant to many other spheres of a learners’ life and work. This includes not only the direct and short-term results in response to learners´ immediate learning needs (eg, improved levels of technical knowledge) but also, as previously reported, it potentially includes additional beneficial long-term contributions suited to the broader needs of learners; for example, the development of more advanced information technology literacy skills, which improves overall work performance, organizational capacity development, and contributes to an individual learner’s continuous professional development and ultimately to the goal of lifelong learning, which is essential to navigate life and work in the 21st century.

A more systematic and nuanced understanding of digital approaches to learning and its impact on a learner’s life is therefore needed [4,6,7]. Thus, OpenWHO’s massive open web-based courses may be considered a paradigm breaker, bringing continuous innovation to pedagogy and learning, in order to provide a truly blended, learner-centered, flexible approach to teaching and learning, which is at the heart of the learning landscape and ecosystem. Other platforms that provide large-scale open-source web-based learning include, for instance, the Indira Gandhi National Open University, which provides a wide range of courses including those related to agriculture, education, and law and has a current total active enrollment of over 4 million students. Moreover, Khan Academy provides web-based courses in mathematics, science, computing, history, economics, and other topics, with more than 10 million users globally subscribing each year.

## Conclusions

This is the first time in the WHO’s history that a learning resource has been launched this rapidly in high-quality, globally accessible learning formats, which are widely and freely available on a massive scale to manage a health threat. The pandemic has shown that web-based learning is no longer a temporary replacement for direct training, but rather a new way for more efficient and equitable learning. The experience and findings reported herein provide guidance for any individual to be better prepared for subsequent instances where a major and fast learning response is required.

## Abbreviations

WHO: World Health Organization
